# Fertility-preservation in endometrial cancer: is it safe? Review of the literature

**DOI:** 10.5935/1518-0557.20160045

**Published:** 2016

**Authors:** Márcia Mendonça Carneiro, Rívia Mara Lamaita, Márcia Cristina França Ferreira, Agnaldo Lopes Silva-Filho

**Affiliations:** 1 Department of Obstetrics and Gynecology, Universidade Federal de Minas Gerais/MG - Brazil; 2 Center for Human Reproduction, Mater Dei Hospital, Belo Horizonte/MG - Brazil

**Keywords:** Assisted reproductive technology, cancer of the endometrium, female infertility, reproductive endocrinology

## Abstract

Almost 5% of women with endometrial cancer are under age 40, and they often have well-differentiated endometrioid estrogen-dependent tumors. Cancer survival rates have improved over the last decades so strategies to avoid or reduce the reproductive damage caused by oncologic treatment are needed. We reviewed the published literature to find evidence to answer the following questions: How should we manage women in reproductive age with endometrial cancer? How safe is fertility preservation in endometrial cancer? Can pregnancy influence endometrial cancer recurrence? What are the fertility sparing options available? Progestins may be prescribed after careful evaluation and counseling. Suitable patients should be selected using imaging methods and endometrial sampling since surgical staging will not be performed. Conservative treatment should only be offered to patients with grade 1 well-differentiated tumors, absence of lymph vascular space invasion, no evidence of myometrial invasion, metastatic disease or suspicious adnexal masses, and expression of progesterone receptors in the endometrium. The presence of co-existing ovarian metastatic of synchronous cancer should be investigated and ruled out before the decision to preserve the ovaries. The availability of Assisted Reproductive Technology (ART) has made it possible for women with endometrial cancer to give birth to a child without compromising their prognoses. Gamete, embryo or ovarian tissue cryopreservation techniques can be employed, although the latter remains experimental. Unfortunately, fertility preservation is rarely considered. Current recommendations for conservative management are based on the overall favorable prognosis of grade 1 minimally invasive tumors. Selected patients with endometrial cancer may be candidates to a safe fertility-preserving management.

## INTRODUCTION

Cancer still represents an enormous global health burden, and published data reveals about 14.1 million new cancer cases, and 8.2 million cancer deaths in 2012 worldwide (Torre *et al*., 2015). Cure remains the most important therapeutic goal and current available therapies are based on surgery, cytotoxic medications and/or radiation. Such procedures unfortunately result in partial or total loss of fertility. Cancer incidence is on the rise worldwide, largely due to the adoption of behaviors and lifestyle factors known to cause cancer such as smoking, aging and growth of the world population (Jemal *et al*., 2011; Torre *et al*., 2015). Cancer survival rates have improved over the last two decades putting quality-of-life issues in the spotlight for women who survive the disease and this includes fertility care (Lee *et al.*, 2006; Jeruss & Woodruff, 2009). The development of assisted reproductive technology (ART) and cryopreservation techniques provided options for female fertility preservation such as oocyte, embryo or ovarian tissue freezing (Lee *et al*., 2006; Rowan, 2010;von Wolff *et al*., 2015; Lambertini *et al*., 2016)

As gynecologic malignancies often affect young women who are still in their reproductive years, and women are postponing childbearing, the incidence of cancer in those who still want to get pregnant has somewhat increased. Rates of permanent infertility and compromised fertility after cancer treatment vary and depend on many factors (Bogani *et al*., 2016; Lambertini *et al*., 2016). The effects of chemotherapy and radiation therapy on fertility depend on a number of factors: the drug or size/location of the radiation field, dose, dose-intensity, method of administration, disease, age, gender, and the pretreatment fertility of the patient (Salama *et al*., 2013; Lawrenz *et al*., 2016). Safe conservative options that preserve fertility are available and may be adopted for those who have not depleted their child-bearing wishes (Rowan, 2010; Levine *et al*., 2015; Druckenmiller *et al*., 2016; Fournier, 2016). New methods for women, such as in vitro follicle maturation and techniques for tissue transplantation, are on the horizon (Loren *et al*., 2013). The FIGO Committee for the Ethical Aspects of Human Reproduction and Women's Health states that cancer treatment is the primary medical goal, and the risks of delaying treatment in order to induce ovarian stimulation and retrieval or ovarian removal or transplant must be carefully considered and should not have a significant impact on treatment (FIGO, 2006).

Endometrial cancer (EC) is the most frequent gynecologic cancer in developed countries killing 34,700 women in 2012 (Torre *et al*., 2015; Bogani *et al*., 2016). Although it is primarily a disease of postmenopausal women, 25% are premenopausal and 3-5% are under age 40 (Zivanovic *et al*., 2009). In this younger group with endometrial cancer a history of ovary dysfunction, anovulation, infertility and obesity are often found. Frequently, these women have never been pregnant and have a strong desire to preserve fertility. In such women endometrial carcinoma is usually an estrogen-dependent well-differentiated endometrioid carcinoma, which does not tend to invade the myometrium and is associated with good prognosis (Benshushan, 2004; Zivanovic *et al.*, 2009; Bogani *et al*., 2016). Therefore, selected patients with endometrial cancer may be candidates to a conservative approach preserving a potential fertility (Carneiro *et al*., 2012).

Recent improvement in the prognosis of cancer patients has drawn attention to fertility issues. Unfortunately, there is a lack of large prospective cohort studies and randomized trials on these topics and, therefore, the safety of such approaches raises concerns among healthcare providers, patients and families. We set out to perform a review of the relevant articles without language restriction based on a PUBMED search using the keywords: "fertility preservation", "endometrial cancer", "surgical treatment", "pregnancy", "chemotherapy" and "radiation". We reviewed the published literature about safe fertility-preserving management in endometrial malignancies, focusing on patient selection criteria, available treatment options and follow-up. We focused on finding evidence to answer the following relevant clinical questions: How should we manage women at reproductive age with endometrial cancer? How safe is fertility preservation in endometrial cancer? Can pregnancy influence endometrial cancer recurrence? What are the fertility sparing options available?

### How should we manage women at reproductive age with endometrial cancer?

The standard treatment for endometrioid carcinoma includes staging laparotomy, total abdominal hysterectomy and bilateral salpingo-oophorectomy with pelvic washing and lymph node sampling when appropriate. The 5-year survival rate after this approach is approximately 94% (Bakkum-Gamez *et al*., 2008). Conservative treatment approaches, with uterine and ovarian preservation may be considered if there is a strong desire to preserve fertility. (Zivanovic *et al.*,2009; Bogani *et al*., 2015). Currently, fertility preservation options in endometrial cancer are limited to hormonal methods (Signorelli *et al*., 2009; Gressel *et al*., 2015). Patients desiring to proceed with conservative hormonal management should be extensively counseled regarding potential risks as no scientifically proven optimal progestin regimen exists (Eskander *et al*., 2011; Loren *et al*., 2013; von Wolff *et al.*, 2015). Response to treatment may vary depending on tumor receptor status, ranging from 26 to 89% in estrogen and progesterone positive tumors but can be as low as 8-17% when these receptors are absent (Chiva *et al*., 2008; Hahn *et al*., 2009; Yu *et al*., 2009).

The conservative treatment of endometrial carcinoma may be recommended when patient desires to preserve fertility, the tumor is endometrioid, its clinical stage is IA FIGO and histological FIGO grade I. It is important to emphasize that such an approach is not standard and should be considered only if the patient insists (Bogani *et al*., 2016; Gressel *et al*., 2015). Careful and thorough counseling is mandatory in this setting (Loren *et al*., 2013; Lambertini *et al.*, 2016). Published data reveals that maintaining the uterus and the ovaries in carefully selected cases with endometrial cancer confers only a very small risk as an increasing number of studies show encouraging results with fertility preserving treatments for endometrial cancer with high dose progestins (Signorelli *et al*., 2009; Rodolakis *et al*., 2015).

Thus, selection of women suitable for such conservative management, as well as treatment options, follow-up, recurrence, obstetric outcomes, and survival rates are vital parameters when counseling these women (Rodolakis *et al*., 2015; von Wolff *et al.,* 2015).

Adequate clinical staging of endometrial cancer remains a challenge while surgical staging is the gold standard. Prognosis is established based on histological grade, depth of myometrial invasion, cervical involvement, vascular space involvement, pelvic and aortic lymph node metastases, adnexal metastases, and positive peritoneal cytology (Guan *et al*., 2011). Apparently, the stage of the tumor is the most important factor in predicting patients' outcome as it determines the mode of treatment and significantly influences survival (Gressel *et al*., 2015; Bogani *et al*., 2016). No optimal method of evaluation prior to conservative management has been identified so far, hence multiple noninvasive or minimally invasive diagnostic methods are employed to attempt to 'clinically stage' a patient (Zivanovic *et al.*, 2009; Bogani *et al*., 2016).

Routine blood and urine exams should be performed and serum levels of CA 125 should be obtained, once its elevated levels suggest advanced disease. Endometrial biopsy is mandatory in the initial evaluation, since histological grade of the tumor is one of the most important prognostic factors (Clarke & Gilks, 2010). To improve the accuracy of clinical staging, different radiological modalities have been used. Transvaginal ultrasound (TVUS), computed tomography (CT) and magnetic resonance imaging (MRI) have been tested and studies revealed no significant difference in their performance. However, contrast-enhanced MRI performed significantly better in the evaluation of the myometrial invasion than non-enhanced MRI, CT or TVUS (*P*<0, 02) (Kinkel *et al*., 1999). When evaluation is inconclusive, thorough laparoscopic exploration with peritoneal cytology, pelvic lymph nodes sampling and adnexal evaluation should be considered before conservative treatment is deployed (Signorelli *et al*., 2009; Bogani *et al*., 2016).

### How safe is fertility preservation in endometrial cancer?

Endometrial carcinoma in patients under the age of 45 is rather uncommon and appears to have more favorable outcome than in older patients (Gressel *et al.*, 2015). Premenopausal women appear to have a higher rate of low-grade tumors and lower stage of disease resulting in a favorable 5-year disease-specific survival rate of 93%, in contrast to older patients (86%) (Crissman *et al*.,1981; Kalogiannidis *et al*., 2011).

Nonetheless, the endometrial carcinoma found at younger ages increases the risk of cancer associated with the Lynch/Hereditary Non-Polyposis Colorectal Cancer (HNPCC) syndrome as well as synchronous or metachronous ovarian cancers occurring outside the setting of Lynch/HNPCC (Evans-Metcalf *et al*., 1998; Richter *et al.,* 2009). In this setting, clinical stage I endometrial carcinoma with metastases to the ovary is rare, comprising only 5% of the cases. The incidence of any stage endometrial carcinoma with a synchronous ovarian malignancy could be as high as 10 to 29.4% (Chiva *et al*., 2008; Navarria *et al*., 2009).

In a study which included 1,365 women with endometrial cancer (Navarria *et al.* 2009), found no significant difference regarding tumor characteristics and survival between young and older patients, except stage of disease (more stage II in the younger group) and rate of synchronous ovarian malignancy (14% in the younger group).

Another study reported a significantly higher rate of ovarian involvement (25%) and recommended prudence when considering ovarian sparing in young endometrial cancer patients with early stage disease (Walsh *et al*., 2005). Richter *et al*. (2009) evaluated 251 patients with endometrial cancer (75.3% stage I) aged 45 or younger. Eleven patients (4.4%) presented with a synchronous serous ovarian malignancy and those submitted to bilateral salpingo-oophorectomy had a significant longer disease-free survival, but no improvement in overall survival. Sun *et al.* (2013) also found that ovarian preservation has no statistically significant impact on the overall survival of young patients with early-stage endometrial cancer.

Ovarian sparing in young patients does not seem to adversely impact the recurrence of early stage endometrial cancer either (Lee *et al*., 2009). One study involving 402 young women with endometrial cancer who underwent hysterectomy with ovarian sparing concluded that, in the absence of risk factors, a conservative approach to surgical staging is feasible, safe and not associated with an increase in cancer-related mortality (Wright *et al*., 2009).

Although endometrial carcinoma is believed to be a hormone-dependent tumor, there is no direct evidence that sparing the ovaries would raise recurrence rates (Lee *et al*., 2009; Sun *et al*., 2013; Wright *et al*., 2016). Ovarian metastases and synchronous primary ovarian cancer in patients with stage I endometrial carcinoma seem to correlate with histological type, depth of myometrial invasion, cervix invasion (including mucosa or/and stroma), uterine serosa extension, fallopian tube involvement, retroperitoneal lymph node metastases, positive peritoneal cytology and CA125 level (Pan *et al*., 2011).

Thus, ovarian preservation at the time of operation, in younger women with stage I endometrial cancer, is worth considering only if ovarian metastasis or synchronous ovarian primary cancer are ruled out. Indeed, the possibility of hidden ovarian metastases call for great caution, especially for patients with high-risk factors (Pan *et al*., 2011; Sun *et al*., 2013). Nevertheless, the ovaries should be preserved in women younger then 45 after a thorough preoperative evaluation and extensive intraoperative exploration. Ovarian preservation apparently had no effect on overall survival and the findings were validated by meta-analysis (Sun *et al*., 2013).

Wright *et al.* (2016) used The National Cancer Database to search for women younger than 50 years of age with stage I endometrioid adenocarcinoma of the endometrium who underwent surgical treatment. The cohort selected 15,648 women: 1,121 (7.2%) who had ovarian preservation and 14,527 (92.8%) who underwent oophorectomy. Data analysis with multivariable models examined predictors of ovarian sparing and the association between ovarian sparing and survival. They concluded that ovarian sparing was not independently associated with survival nor there was an association between ovarian preservation and survival. Unfortunately, despite these reassuring data, the majority of young women with endometrial cancer still undergo oophorectomy.

### Can pregnancy influence endometrial cancer recurrence?

It is very important to emphasize the need to discuss with the patient the risks of conservative treatment. Although the degree of histological differentiation is a sensitive indicator of tumor spread, 2.8% of all grade 1 lesions have pelvic node involvement, and 1.7% bear para-aortic node involvement. Moreover, 10% of grade 1 tumors have deep muscle invasion, 6% of clinical stage I and hidden stage II patients have spread of tumor to the adnexa and 19% of patients have coexisting ovarian neoplasm (Crissman *et al*., 1981).

Progestin therapy remains the most common option when fertility-sparing is considered, as it is highly effective in selected cases. Various doses of different progestational agents have been used in an effort to preserve fertility in patients with clinical stage I endometrial carcinoma. Oral medroxyprogesterone acetate (MPA) at a dose of 100-800 mg/day; megestrol acetate (MA) at a dose of 40-160 mg/day and a combination of tamoxifen and a progestin have been used with similar results (Zhou *et al*., 2015; Inoue *et al.*, 2016). The follow-up of these patients under conservative treatment in the first year included serial TVUS, endometrial biopsy and CA-125. Periodic endometrial samplings should be performed every 1 to 6 months. Close follow-up during and after the period treatment is strictly recommended. (Pronin *et al*., 2015;.Park & Nam, 2015).

Fung-Kee-Fung (2006) published a systematic review of sixteen non-comparative retrospective studies in an attempt to establish the optimum follow-up for women treated with potentially curative treatment for endometrial cancer. Routine testing seems to be of limited benefit for patients at low risk of disease since most recurrences occur within 3 years in high risk patients, and involve symptoms (Fung-Kee-Fung *et al*., 2006; Mazzon *et al*., 2010).

To date, the time required for response to conservative treatment and its duration have not been established. Published data reveals that the minimal time to response was 3.6 months and the treatment was maintained for 5.4 months (Gotlieb *et al*., 2003). Although today there is no consensus as to which progestational agent to use, or treatment dose and length, it appears that 62-75% of women with clinical stage I and well differentiated adenocarcinoma respond well to progestational treatment within 3 to 9 months and the majority will have long term response (Pronin *et al*., 2015;.Park & Nam, 2015). As long as an accurate pretreatment assessment is performed, progestin therapy is an appropriate option to preserve fertility in young women with well-differentiated endometrial carcinoma or severe atypical endometrial hyperplasia (Pronin *et al*., 2015; Inoue *et al*., 2016). The absence of progesterone receptors (PR), however, can jeopardize the success of progestin as a treatment (Yang *et al*., 2005). Nevertheless, there is no need to check for PR expression routinely, because a significant number of PR negative tumors will respond to treatment (Rodokakis *et al*., 2015).

Eskander *et al*. (2011) recommend that candidates to hormonal fertility-sparing treatment should fulfill the following criteria: (1) grade 1 well-differentiated tumor; (2) absence of lymph vascular space invasion (LVSI) on adequate curettage specimen; (3) no evidence of myometrial invasion on MRI; (4) no evidence of metastatic disease on CT imaging; (5) no evidence of a suspicious adnexal mass on CT or TVUS; and (6) strong and diffuse immunohistochemical expression of progesterone receptors on endometrial biopsy or curettage specimen.

The overall response rate, evaluated by endometrial biopsy every three months, to either medroxyprogesterone acetate or megestrol acetate was 73% in a median time of 4 months (range 1-15 months). The relapse rate was 36% in a median follow-up time of 22 months (range 6-73 months). Overall, 40% of patients who responded successfully, conceived; half of them using assisted reproductive technology (ART) so as to achieve an immediate pregnancy (Kalogiannidis &, Agorastos, 2011).

There are reports of many pregnancies after conservative management of endometrial carcinoma, some after ART (Fujimoto *et al*., 2014; Koskas *et al*., 2014) Combining conservative treatment with ART may result in healthy infants without an adverse effect on oncologic prognosis (Elizur *et al.*, 2007; Bozdag *et al.,* 2009; Mao *et al*.,2010). Introduction of infertility treatment ART soon after achieving tumor remission by MPA would be beneficial for patients in this setting. Although preliminary results are encouraging, the majority of the series reported so far are retrospective, included only a small number of patients, and used different treatment methods and inclusion criteria, making the extraction of useful conclusions rather difficult (Koskas *et al*., 2014; Rodolakis *et al*., 2015; Inoue *et al.*, 2016). The recommendations of the European Society of Gynecological Oncology Task Force for Fertility Preservation state that MPA or MA are the progestins to be used as more studies are needed to further elucidate the role of the levonorgestrel intrauterine device (LNG-IUD) (Rodolakis *et al*., 2015).

### What are the available strategies of fertility preservation?

For patients planning to have chemotherapy, radiotherapy or scheduled to undergo bilateral oophorectomy, the loss of ovarian function will result in premature ovarian failure and permanent loss of fertility. Potential strategies for such women include embryo or oocyte cryopreservation (Gressel *et al*., 2015; Zapardiel *et al*., 2016). However, embryo cryopreservation is not suitable for children and unmarried women as it involves a male partner, unless sperm donation is acceptable (Zapardiel *et al*., 2016). Embryo cryopreservation also requires superovulation, which is time consuming and not without side effects. Cancer patients respond to gonadotropins but stimulation lasts longer and a higher total dose is required. No significant differences in the number of oocytes retrieved, matured oocytes and the fertilization rate were found (Knopman *et al*., 2009).

The safety of ART in women with a past history of gynaecological cancer raises concerns, though some studies report reassuring data. Ovulation induction does not appear to be associated with increased risk of relapse, and subsequent pregnancies do not worsen oncological outcomes (Matthews *et al*., 2012; Fujimoto *et al*., 2014; Zapardiel *et al*., 2016). The impact of high serum estradiol levels on endometrial carcinoma is uncertain, although some data suggest an adverse effect of ovarian stimulation. It seems that there is no clearly optimal duration, protocol or number of attempts for ovarian stimulation in women with early-stage endometrial carcinoma (Zapardiel *et al*., 2016).

There are strategies to keep estrogen levels low during controlled ovarian hyperstimulation (COH) so that estrogen-dependent cancer patients are safe and cancer recurrence is not increased (Oktay *et al*., 2010). Studies involving breast cancer patients revealed that the use of aromatase-inhibitors combined with a gonadotropin-releasing hormone agonist (GNRHa) to trigger ovulation, instead of human chorionic gonadotropin (hCG), may reduce estrogen exposure and the incidence of Ovarian Hyperstimulation Syndrome. GnRHa ovulation trigger resulted in a larger number and higher percentage of mature oocytes and a higher number of cryopreserved embryos or oocytes compared with hCG cycles (Oktay *et al*., 2010). Recent evidence also indicates that there are multiple main follicle recruitment waves during the menstrual cycle and hence the concept of a narrow window of opportunity for follicle recruitment may not be accurate. Therefore, the current availability of GnRH antagonists combined with multiple recruitment waves allows the beginning of random-start controlled ovarian hyperstimulation (COH) in the late follicular or luteal phase of the menstrual cycle for embryo cryopreservation in cancer patients. Unfortunately, published data regarding late-follicular or luteal-start COH and emergency fertility sparing is still limited to case series (Sönmezer *et al*., 2011; Kreskin *et al*., 2014).

The American Society for Reproductive Medicine (ASRM) has recently reviewed the evidence on fertilization and pregnancy rates obtained after oocyte vitrification and warming. Published data, although limited, shows results that are similar to those obtained when fresh oocytes are used in IVF/ICSI cycles. As for chromosomal abnormalities, birth defects and developmental alterations, there is no increase in comparison to pregnancies after conventional IVF/ICSI and the general population. Therefore, the ASRM recommends that oocyte vitrification and warming should no longer be regarded as experimental (ASRM & SART, 2013), a decision endorsed by the American College of Obstetricians and Gynecologists' Committee on Gynecologic Practice (ACOG, 2014). Available data is still scant to recommend oocyte cryopreservation for the sole purpose of circumventing reproductive aging in otherwise healthy women.

Results from clinical trials and observational studies show that the cryopreservation of unfertilized oocytes represents an acceptable and often viable alternative, particularly for single women, and that it should be offered as a routine technique for female patients before chemo and/or radiotherapy (Noyes *et al*., 2010; Noyes *et al*., 2011; Cobo *et al.*, 2011). Apparently, fresh and frozen oocytes result in comparable pregnancy rates in IVF cycles, endorsing the use of such technologies in well-selected patients aged 35 years and younger (Cobo *et al*., 2011; Garcia-Velasco *et al*., 2013; ACOG, 2014).

In spite of being a standardized technique, results after oocyte vitrification vary depending on a host of variables including the specific population, methodologies applied, particular protocols, types of device and cryoprotectants. Although protocols may seem simple to use, success relies on the availability of experienced hands in the laboratory (Cobo *et al*., 2013).

The safety of the technique can be assessed looking at 936 babies born from frozen oocytes from multiple centers around the world with no apparent increase in the rate of congenital anomalies (Noyes *et al*., 2009). Oocyte vitrification appears to be an efficient method to preserve oocytes, regarding oocyte survival, fertilization, embryo development and pregnancy rates as well as neonatal data, but further large controlled clinical trials are needed to corroborate such early reassuring outcomes (ASRM & SART, 2013; Garcia-Velasco *et al*., 2013; ACOG, 2014).

Ovarian tissue cryopreservation is a promising strategy that offers the possibility to restore fertility by autotransplantation or in vitro culture and oocyte maturation (Ledda *et al*., 2001). It offers the advantages of enabling the storage of a large number of gametes and be rapidly performed, at any period of the cycle, without delaying the oncological treatment (Lotz *et al*., 2016). However, several considerations involve the creation of a bank of frozen ovarian tissue and, although several protocols of slow freezing and fast thawing showed exciting results, the real consequences of cryopreservation and the ideal protocol remain uncertain. In addition, ovarian tissue cryopreservation is still considered experimental (Salama & Woodruff, 2015).

Autotransplantation of ovarian tissue has yielded 60 live births to date, including one from tissue that was cryostored in adolescence. Advantages include immediate initiation of oncologic treatment, ability to restore physiological ovarian function and no need for ovarian hyperstimulation. In addition, it may be the only option for fertility preservation for prepubertal girls or young women with estrogen-sensitive cancers. However, it is assumed that autografting cryopreserved-thawed ovarian cortical tissue poses a risk of reseeding the malignancy (Salama & Woodruff, 2015; Abir *et al*., 2016).

Technical difficulties and the complex human folliculogenesis process will probably delay the development of in vitro culture systems to support human primordial follicular growth until the ovulatory stage (Salama & Woodruff, 2015).

After transplantation, follicular development and restoration of hormone secretion have been investigated in animal and human studies (Torrents *et al*., 2003). In humans, since the first live birth after autotransplantation of cryopreserved ovarian tissue reported in 2004, ovarian cortex transplantation has led to the birth of 60 healthy babies, and one pregnancy after IVF (Donnez *et al*., 2013; Donnez & Dolmans, 2015).

Imbert *et al.* (2014) retrospectively analyzed ovarian function and fertility recovery rates, as well as ovarian tissue characteristics, of 225 women who underwent ovarian tissue cryopreservation. Ovarian function returned in 71 post-pubertal patients without the need for grafts of cryopreserved tissue. Thirty-three spontaneous pregnancies were reported, leading to 34 live births. Among the 13 pre-pubertal patients who reached pubertal age during the follow-up, 10 had premature ovarian failure (POF). Eight patients received cryopreserved ovarian grafts to reverse POF and three of them had already become pregnant. Dittrich *et al*. (2015) also reported the results of 20 orthotopic retransplantations of cryopreserved ovarian tissue after cancer treatment. Ovarian activity resumed in all patients except one. Seven patients conceived, with one miscarriage and four ongoing pregnancies.

Data published on the restoration of ovarian function, pregnancies and live birth rates suggests that preserving fertility by cryopreserving ovarian tissue is a successful and safe clinical option that can be considered for selected cancer patients (Imbert *et al*., 2014; ASRM, 2014; Dittrich *et al*., 2015). The ASRM (2014) stresses that ovarian tissue cryopreservation and subsequent transplantation can only be recommended as an experimental protocol in carefully selected patients. It should not be offered with the intent to delay pregnancy or for benign conditions in that there is a potential risk of reintroducing malignancy.

Non-invasive techniques have been used in an attempt to minimize the gonadotoxic effect of chemotherapy by using GnRHa or oral contraceptives (OC) to stop the maturation of the dividing oocyte, producing its involution and avoiding the noxious effect of the chemotherapy on the dividing cell (Blumenfeld & von Wolff, 2008). Studies have shown an 11.1% incidence of premature ovarian failure in patients who received GnRH-a, compared with a 55.5% incidence in the controls. Others argue that there is absence of conclusive evidence regarding the safety and efficacy of GnRHa treatment in protecting against chemotherapy-induced gonadal injury (Blumenfeld *et al*., 2007; Badawy *et al*., 2009; Gerber *et al*., 2011). Possible mechanisms of action include reduction of the number of primordial follicles entering the differentiation stage, diminished ovarian perfusion and delivery of chemotherapy to the ovaries, and maybe a direct effect with on the upregulation of an intragonadal antiapoptotic molecule (Blumenfeld & von Wolff, 2008). Nevertheless, the available evidence is still limited on the fertility preserving effect of OC. Two studies showed lower premature ovarian failure rates in OC-treated patients: 13.2% compared with 29.8% among the controls (Blumenfeld & von Wolff, 2008).

Controversy remains regarding the use of gonadotrophin releasing hormone agonist (GnRH-a) or combined oral contraceptive administered at time of the gonadotoxic therapy to prevent premature ovarian failure in women. The available published data from both human and animal studies show mixed results. The best way to preserve fertility and ovarian function in young women undergoing chemotherapy still remains to be determined. In the absence of a consensus, each case should be carefully evaluated, considering the patient's wishes and expectations, the type of chemotherapy, age, obstetric history, ovarian reserve (combining multiple indicators such as basal hormone profile, anti müllerian hormone -AMH- and antral follicle count), as well as family history of premature ovarian failure (Chahvar *et al*., 2014). Currently, the ASCO guideline (ASCO, 2013) reports that there is insufficient evidence regarding the effectiveness of GnRHa and other means of ovarian suppression in fertility preservation.

## FINAL CONSIDERATIONS

In spite of the importance of this topic, fertility preservation methods are still relatively infrequently applied in the cancer population, limiting the development of knowledge on the success and effects of different interventions. The American Society of Clinical Oncology highlights that the fertility preservation literature reveals a paucity of large and/or randomized studies.

Current recommendations for conservative management are based on the overall favorable prognosis of grade 1 minimally invasive tumors, supported by a few case series and case reports, but no prospective data. Selected patients with endometrial cancer may be candidates to a safe fertility-preserving management strategy. Two issues are extremely relavant when a conservative approach is considered: first, the evaluation of the tumor's individual pathology biology (histological type, grade, myometrial invasion, and presence of lymphovascular space invasion); and second, choosing the optimal approaches for fertility sparing and follow up. Large multicentric trials are needed so as to define the best selection criteria for a conservative treatment, endocrine regimen of choice, optimal dosing, duration and follow-up protocols.

## References

[r1] Abir R, Ben-Aharon I, Garor R, Yaniv I, Ash S, Stemmer SM, Ben-Haroush A, Freud E, Kravarusic D, Sapir O, Fisch B (2016). Cryopreservation of in vitro matured oocytes in addition to
ovarian tissue freezing for fertility preservation in paediatric female
cancer patients before and after cancer therapy. Hum Reprod.

[r2] ACOG - American College of Obstetricians and Gynecologists (2014). Oocyte cryopreservation. ACOG:Committee Opinion No.
584. Obstet Gynecol.

[r3] SRM - The Practice Committees of American Society for Reproductive
Medicine, SART - Society for Assisted Reproductive Technology (2013). Mature oocyte cryopreservation: a guideline. Fertil Steril.

[r4] ASRM (2014). Practice Committee of American Society for Reproductive Medicine
Ovarian tissue cryopreservation: a committee opinion. Fertil Steril.

[r5] Badawy A, Elnashar A, El-Ashry M, Shahat M (2009). Gonadotropin-releasing hormone agonists for prevention of
chemotherapy-induced ovarian damage: prospective randomized
study. Fertil Steril.

[r6] Bakkum-Gamez JN, Gonzalez-Bosquet J, Laack NN, Mariani A, Dowdy SC (2008). Current issues in the management of endometrial
cancer. Mayo Clin Proc.

[r7] Blumenfeld Z, von Wolff M (2008). GnRH-analogues and oral contraceptives for fertility preservation
in women during chemotherapy. Hum Reprod Update.

[r8] Blumenfeld Z (2007). How to preserve fertility in young women exposed to chemotherapy?
The role of GnRH agonist cotreatment in addition to cryopreservation of
embryos oocytes, or ovaries. Oncologist.

[r9] Bogani G, Dowdy SC, Cliby WA, Ghezzi F, Rossetti D, Frigerio L, Mariani A (2016). Management of endometrial cancer: issues and
controversies. Eur J Gynaecol Oncol.

[r10] Bozdag G, Yarali H, Polat M, Esinler I, Tiras B, Ayhan A (2009). ICSI outcome following conservative fertility sparing management
of endometrial cancer. Reprod Biomed Online.

[r11] Carneiro MM, Lamaita RM, Ferreira MCF, Silva-Filho AL (2012). Safe Fertility-Preserving Management in Endometrial Cancer: Is It
Feasible?. Review of the Literature J Gynecol Surg.

[r12] Chahvar ST, Al-Shawaf T, Tranquilli AL (2014). Pharmacologic ovarian preservation in young women undergoing
chemotherapy. Curr Med Chem.

[r13] Chiva L, Lapuente F, González-Cortijo L, Carballo N, García JF, Rojo A, Gonzalez-Martín A (2008). Sparing fertility in young patients with endometrial
cancer. Gynecol Oncol.

[r14] Clarke BA, Gilks CB (2010). Endometrial carcinoma: controversies in histopathological
assessment of grade and tumour cell type. J Clin Pathol.

[r15] Cobo A, Diaz C (2011). Clinical application of oocyte vitrification: a systematic review
and meta-analysis of randomized controlled trials. Fertil Steril.

[r16] Cobo A, Garcia-Velasco JA, Domingo J, Remohí J, Pellicer A (2013). Is vitrification of oocytes useful for fertility preservation for
age-related fertility decline and in cancer patients?. Fertil Steril.

[r17] Crissman JD, Azoury RS, Barners AE, Schellhas HF (1981). Endometrial cancer in women 40 years age or
younger. Obstet Gynecol.

[r18] Dittrich R, Hackl J, Lotz L, Hoffmann I, Beckmann MW (2015). Pregnancies and live births after 20 transplantations of
cryopreserved ovarian tissue in a single center. Fertil Steril.

[r19] Donnez J, Dolmans MM, Pellicer A, Diaz-Garcia C, Sanchez Serrano M, Schmidt KT, Ernst E, Luyckx V, Andersen CY (2013). Restoration of ovarian activity and pregnancy after
transplantation of cryopreserved ovarian tissue: a review of 60 cases of
reimplantation. Fertil Steril.

[r20] Donnez J, Dolmans MM (2015). Ovarian cortex transplantation: 60 reported live births brings
the success and worldwide expansion of the technique towards routine
clinical practice. J Assist Reprod Genet.

[r21] Druckenmiller S, Goldman KN, Labella PA, Fino ME, Bazzocchi A, Noyes N (2016). Successful Oocyte Cryopreservation in Reproductive-Aged Cancer
Survivors. Obstet Gynecol.

[r22] Elizur SE, Beiner ME, Korach J, Weiser A, Ben-Baruch G, Dor J (2007). Outcome of in vitro fertilization treatment in infertile women
conservatively treated for endometrial adenocarcinoma. Fertil Steril.

[r23] Eskander RN, Randall LM, Berman ML, Tewari KS, Disaia PJ, Bristow RE (2011). Fertility preserving options in patients with gynecologic
malignancies. Am J Obstet Gynecol.

[r81] Evans-Metcalf ER, Brooks SE, Reale FR, Baker SP (1998). Profile of women 45 years of age and younger with endometrial
cancer. Obstet Gynecol.

[r24] FIGO Committee for the Ethical Aspects of Human Reproduction and
Women's Health (2006). Ethical considerations and recommendations on oocyte and ovarian
cryopreservation. Int J Gynaecol Obstet.

[r25] Fournier EM (2016). Oncofertility and the Rights to Future Fertility. JAMA Oncol.

[r26] Fujimoto A, Ichinose M, Harada M, Hirata T, Osuga Y, Fujii T (2014). The outcome of infertility treatment in patients undergoing
assisted reproductive technology after conservative therapy for endometrial
cancer. J Assist Reprod Genet.

[r27] Fung-Kee-Fung M, Dodge J, Elit L, Lukka H, Chambers A, Oliver T, Cancer Care Ontario Program in Evidence-based Care Gynecology Cancer
Disease Site Group (2006). Follow-up after primary therapy for endometrial cancer: a
systematic review. Gynecol Oncol.

[r28] Garcia-Velasco JA, Domingo J, Cobo A, Martínez M, Carmona L, Pellicer A (2013). Five years' experience using oocyte vitrification to preserve
fertility for medical and nonmedical indications. Fertil Steril.

[r29] Gerber B, von Minckwitz G, Stehle H, Reimer T, Felberbaum R, Maass N, Fischer D, Sommer HL, Conrad B, Ortmann O, Fehm T, Rezai M, Mehta K, Loibl S, German Breast Group Investigators (2011). Effect of Luteinizing Hormone-Releasing Hormone Agonist on
Ovarian Function After Modern Adjuvant Breast Cancer Chemotherapy: The GBG
37 ZORO Study. J Clin Oncol.

[r30] Gotlieb WH, Beiner ME, Shalmon B, Korach Y, Segal Y, Zmira N, Koupolovic J, Ben-Baruch G (2003). Outcome of fertility-sparing treatment with progestins in young
patients with endometrial cancer. Obstet Gynecol.

[r31] Gressel GM, Parkash V, Pal L (2015). Management options and fertility-preserving therapy for
premenopausal endometrial hyperplasia and early-stage endometrial
cancer. Int J Gynaecol Obstet.

[r32] Guan H, Semaan A, Bandyopadhyay S, Arabi H, Feng J, Fathallah L, Pansare V, Qazi A, Abdul-Karim F, Morris RT, Munkarah AR, Ali-Fehmi R (2011). Prognosis and reproducibility of new and existing binary grading
systems for endometrial carcinoma compared to FIGO grading in hysterectomy
specimens. Int J Gynecol Cancer.

[r33] Hahn HS, Yoon SG, Hong JS (2009). Conservative treatment with progestin and pregnancy outcomes in
endometrial cancer. Int J Gynecol Cancer.

[r34] Imbert R, Moffa F, Tsepelidis S, Simon P, Delbaere A, Devreker F, Dechene J, Ferster A, Veys I, Fastrez M, Englert Y, Demeestere I (2014). Safety and usefulness of cryopreservation of ovarian tissue to
preserve fertility: a 12-year retrospective analysis. Hum Reprod.

[r35] Inoue O, Hamatani T, Susumu N, Yamagami W, Ogawa S, Takemoto T, Hirasawa A, Banno K, Kuji N, Tanaka M, Aoki D (2016). Factors affecting pregnancy outcomes in young women treated with
fertility-preserving therapy for well-differentiated endometrial cancer or
atypical endometrial hyperplasia. Reprod Biol Endocrinol.

[r36] Jemal A, Bray F, Center MM, Ferlay J, Ward E, Forman D (2011). Global cancer statistics. CA Cancer J Clin.

[r37] Jeruss JS, Woodruff TK (2009). Preservation of fertility in patients with cancer. N Engl J Med.

[r38] Kalogiannidis I, Agorastos T (2011). Conservative management of young patients with endometrial
highly-differentiated adenocarcinoma. J Obstet Gynaecol.

[r39] Keskin U, Ercan CM, Yilmaz A, Babacan A, Korkmaz C, Duru NK, Ergun A (2014). Random-start controlled ovarian hyperstimulation with letrozole
for fertility preservation in cancer patients: case series and review of
literature. J Pak Med Assoc.

[r40] Kinkel K, Kaji Y, Yu KK, Segal MR, Lu Y, Powell CB, Hricak H (1999). Radiologic staging in patients with endometrial cancer: a
meta-analysis. Radiology.

[r41] Knopman JM, Noyes N, Talebian S, Krey LC, Grifo JA, Licciardi F (2009). Women with cancer undergoing ART for fertility preservation: a
cohort study of their response to exogenous gonadotropins. Fertil Steril.

[r42] Koskas M, Uzan J, Luton D, Rouzier R, Daraï E (2014). Prognostic factors of oncologic and reproductive outcomes in
fertility-sparing management of endometrial atypical hyperplasia and
adenocarcinoma: systematic review and meta-analysis. Fertil Steril.

[r43] Lambertini M, Del Mastro L, Pescio MC, Andersen CY, Azim Jr HA, Peccatori FA, Costa M, Revelli A, Salvagno F, Gennari A, Ubaldi FM, La Sala GB, De Stefano C, Wallace WH, Partridge AH, Anserini P (2016). Cancer and fertility preservation: international recommendations
from an expert meeting. BMC Med.

[r44] Lawrenz B, Mahajan N, Fatemi HM (2016). The effects of cancer therapy on women's fertility: what do we
know now?. Future Oncol.

[r45] Ledda S, Lei G, Bogliolo L, Naitana S (2001). Oocyte cryopreservation and ovarian tissue
banking. Theriogenology.

[r46] Lee SJ, Schover LR, Partridge AH, Patrizio P, Wallace WH, Hagerty K, Beck LN, Brennan LV, Oktay K (2006). American Society of Clinical Oncology. J Clin Oncol.

[r47] Lee TS, Kim JW, Kim TJ, Cho CH, Ryu SY, Ryu HS, Kim BG, Lee KH, Kim YM, Kang SB, Korean Gynecologic Oncology Group (2009). Ovarian preservation during the surgical treatment of early stage
endometrial cancer: a nation-wide study conducted by the Korean Gynecologic
Oncology Group. Gynecol Oncol.

[r48] Levine JM, Kelvin JF, Quinn GP, Gracia CR (2015). Infertility in reproductive-age female cancer
survivors. Cancer.

[r49] Loren AW, Mangu PB, Beck LN, Brennan L, Magdalinski AJ, Partridge AH, Quinn G, Wallace WH, Oktay K, American Society of Clinical Oncology (2013). Fertility preservation for patients with cancer: American Society
of Clinical Oncology clinical practice guideline update. J Clin Oncol.

[r50] Lotz L, Maktabi A, Hoffmann I, Findeklee S, Beckmann MW1, Dittrich R (2016). Ovarian tissue cryopreservation and retransplantation - what do
patients think about it?. Reprod Biomed Online.

[r51] Mao Y, Wan X, Chen Y, Lv W, Xie X (2010). Outcomes of conservative therapy for young women with early
endometrial adenocarcinoma. Fertil Steril.

[r52] Matthews ML, Hurst BS, Marshburn PB, Usadi RS, Papadakis MA, Sarantou T (2012). Cancer, fertility preservation, and future pregnancy: a
comprehensive review. Obstet Gynecol Int.

[r53] Mazzon I, Corrado G, Masciullo V, Morricone D, Ferrandina G, Scambia G (2010). Conservative surgical management of stage IA endometrial
carcinoma for fertility preservation. Fertil Steril.

[r54] Navarria I, Usel M, Rapiti E, Neyroud-Caspar I, Pelte MF, Bouchardy C, Petignat P (2009). Young patients with endometrial cancer: how many could be
eligible for fertility-sparing treatment?. Gynecol Oncol.

[r55] Noyes N, Knopman JM, Long K, Coletta JM, Abu-Rustum NR (2011). Fertility considerations in the management of gynecologic
malignancies. Gynecol Oncol.

[r56] Noyes N, Labella PA, Grifo J, Knopman JM (2010). Oocyte cryopreservation: a feasible fertility preservation option
for reproductive age cancer survivors. J Assist Reprod Genet.

[r57] Noyes N, Porcu E, Borini A (2009). Over 900 oocyte cryopreservation babies born with no apparent
increase in congenital anomalies. Reprod Biomed Online.

[r58] Oktay K, Türkçüoğlu I, Rodriguez-Wallberg KA (2010). GnRH agonist trigger for women with breast cancer undergoing
fertility preservation by aromatase inhibitor/FSH
stimulation. Reprod Biomed Online.

[r59] Pan Z, Wang X, Zhang X, Chen X, Xie X (2011). Retrospective analysis on coexisting ovarian cancer in 976
patients with clinical stage I endometrial carcinoma. J Obstet Gynaecol Res.

[r60] Park JY, Nam JH (2015). Progestins in the fertility-sparing treatment and retreatment of
patients with primary and recurrent endometrial cancer. Oncologist.

[r61] Pronin SM, Novikova OV, Andreeva JY, Novikova EG (2015). Fertility-Sparing Treatment of Early Endometrial Cancer and
Complex Atypical Hyperplasia in Young Women of Childbearing
Potential. Int J Gynecol Cancer.

[r62] Richter CE, Qian B, Martel M, Yu H, Azodi M, Rutherford TJ, Schwartz PE (2009). Ovarian preservation and staging in reproductive-age endometrial
cancer patients. Gynecol Oncol.

[r63] Rodolakis A, Biliatis I, Morice P, Reed N, Mangler M, Kesic V, Denschlag D (2015). European Society of Gynecological Oncology Task Force for
Fertility Preservation: Clinical Recommendations for Fertility-Sparing
Management in Young Endometrial Cancer Patients. Int J Gynecol Cancer.

[r64] Rowan K (2010). Fertility preservation during treatment is a growing issue for
women. J Natl Cancer Inst.

[r65] Salama M, Winkler K, Murach KF, Seeber B, Ziehr SC, Wildt L (2013). Female fertility loss and preservation: threats and
opportunities. Ann Oncol.

[r66] Salama M, Woodruff TK (2015). New advances in ovarian autotransplantation to restore fertility
in cancer patients. Cancer Metastasis Rev.

[r67] Signorelli M, Caspani G, Bonazzi C, Chiappa V, Perego P, Mangioni C (2009). Fertility-sparing treatment in young women with endometrial
cancer or atypical complex hyperplasia: a prospective single-institution
experience of 21 cases. BJOG.

[r68] Sönmezer M, Türkçüoğlu I, Coşkun U, Oktay K (2011). Random-start controlled ovarian hyperstimulation for emergency
fertility preservation in letrozole cycles. Fertil Steril.

[r69] Sun C, Chen G, Yang Z, Jiang J, Yang X, Li N, Zhou B, Zhu T, Wei J, Weng D, Ma D, Wang C, Kong B (2013). Safety of ovarian preservation in young patients with early-stage
endometrial cancer: a retrospective study and meta-analysis. Fertil Steril.

[r70] Torre LA, Bray F, Siegel RL, Ferlay J, Lortet-Tieulent J, Jemal A (2015). Global cancer statistics, 2012. CA Cancer J Clin.

[r71] Torrents E, Boiso I, Barri PN, Veiga A (2003). Applications of ovarian tissue transplantation in experimental
biology and medicine. Hum Reprod Update.

[r72] von Wolff M, Dittrich R, Liebenthron J, Nawroth F, Schüring AN, Bruckner T, Germeyer A (2015). Fertility-preservation counselling and treatment for medical
reasons: data from a multinational network of over 5000
women. Reprod Biomed Online.

[r73] Walsh C, Holschneider C, Hoang Y, Tieu K, Karlan B, Cass I (2005). Coexisting ovarian malignancy in young women with endometrial
cancer. Obstet Gynecol.

[r74] Wright JD, Buck AM, Shah M, Burke WM, Schiff PB, Herzog TJ (2009). Safety of ovarian preservation in premenopausal women with
endometrial cancer. J Clin Oncol.

[r75] Wright JD, Jorge S, Tergas AI, Hou JY, Burke WM, Huang Y, Hu JC, Ananth CV, Neugut AI, Hershman DL (2016). Utilization and Outcomes of Ovarian Conservation in Premenopausal
Women With Endometrial Cancer. Obstet Gynecol.

[r76] Yang YC, Wu CC, Chen CP, Chang CL, Wang KL (2005). Reevaluating the safety of fertility-sparing hormonal therapy for
early endometrial cancer. Gynecol Oncol.

[r77] Yu M, Yang JX, Wu M, Lang JH, Huo Z, Shen K (2009). Fertility-preserving treatment in young women with
well-differentiated endometrial carcinoma and severe atypical hyperplasia of
endometrium. Fertil Steril.

[r78] Zapardiel I, Cruz M, Diestro MD, Requena A, Garcia-Velasco JA (2016). Assisted reproductive techniques after fertility-sparing
treatments in gynaecological cancers. Hum Reprod Update.

[r79] Zhou R, Yang Y, Lu Q, Wang J, Miao Y, Wang S, Wang Z, Zhao C, Wei L (2015). Prognostic factors of oncological and reproductive outcomes in
fertility-sparing treatment of complex atypical hyperplasia and low-grade
endometrial cancer using oral progestin in Chinese patients. Gynecol Oncol.

[r80] Zivanovic O, Carter J, Kauff ND, Barakat RR (2009). A review of the challenges faced in the conservative treatment of
young women with endometrial carcinoma and risk of ovarian
cancer. Gynecol Oncol.

